# Structure-Guided Discovery of a Cold-Responsive Antifreeze-like Protein from Antarctic *Flavobacterium* sp. PL002

**DOI:** 10.3390/microorganisms14071571

**Published:** 2026-07-17

**Authors:** Jennifer Charles Labo, Hui Yin Fan, Hackwon Do, Hoang Thi Ngoc Trang, Paris Lavin, Mohd Faizal Abu Bakar, Nur Athirah Yusof

**Affiliations:** 1 Biotechnology Research Institute, Universiti Malaysia Sabah, Kota Kinabalu 88450, Sabah, Malaysia; 2 Food Security Research Laboratory, Faculty of Food Science and Nutrition, Universiti Malaysia Sabah, Kota Kinabalu 88400, Sabah, Malaysia; 3 Research Unit for Low-Temperature Convergence Technology, Korea Polar Research Institute, Incheon 21990, Republic of Korea; 4 Department of Polar Sciences, University of Science and Technology, Incheon 21990, Republic of Korea; 5 Departamento de Biotecnologia, Facultad de Ciencias del Mar y Recursos Biologicos, Universidad de Antofagasta, 601 Avenida Angamos, Antofagasta 1270300, Chile; 6 Advanced Genomics and Bioinformatics, Malaysia Genome and Vaccine Institute, National Institutes of Biotechnology Malaysia, Jalan Bangi, Kajang 43000, Selangor, Malaysia

**Keywords:** Antarctic microorganisms, *Flavobacterium*, antifreeze-like protein, ice-binding protein, ice recrystallisation inhibition, cold adaptation, hypothetical proteins

## Abstract

Antarctic microorganisms experience persistent subzero temperatures and repeated freeze–thaw cycles that require specialized mechanisms for survival. Although transcriptomic studies have identified numerous cold-responsive genes, many remain annotated as hypothetical proteins with unknown functions. In this study, we investigated PL002-1792, a strongly upregulated hypothetical protein from Antarctic *Flavobacterium* sp. PL002 identified under severe cold stress (−20 °C versus −6 °C; log_2_ fold change = 5.61, adjusted *p* = 1.89 × 10^−138^). Sequence analysis revealed a 402-amino-acid protein containing a predicted Sec/SPII lipoprotein signal peptide. AlphaFold3 prediction generated a high-confidence structural model (pTM = 0.96) with an elongated β-sheet-rich architecture resembling bacterial ice-binding proteins. Comparative analysis with the ice-binding protein from *Flavobacterium frigoris* (*FfIBP*) identified conserved glycine-rich and TXT-like motifs associated with putative ice-binding surfaces. Recombinant PL002-1792 was expressed in *Escherichia coli*, recovered from inclusion bodies, and successfully refolded into a predominantly β-sheet-rich conformation as confirmed by circular dichroism spectroscopy. Functional assays demonstrated moderate ice recrystallisation inhibition activity, reducing relative ice crystal mean grain size to approximately 90% of the control, and significantly enhanced freeze–thaw survival, with recombinant cells retaining 67% viability after three freeze–thaw cycles compared with 27% for the empty-vector control. These findings identify PL002-1792 as a novel antifreeze-like protein and highlight the utility of structure-guided approaches for uncovering previously uncharacterized cold-adaptation mechanisms in polar microorganisms.

## 1. Introduction

Antarctic environments represent some of the most extreme ecosystems on Earth, characterized by persistent subzero temperatures, repeated freeze–thaw cycles, high salinity in sea-ice habitats, and limited nutrient availability [1]. These conditions impose severe physiological stress on microorganisms, requiring specialized adaptations to maintain cellular integrity and metabolic activity. One of the most significant challenges associated with subzero environments is the formation and recrystallisation of ice. Ice formation around microbial cells can restrict oxygen diffusion, impair nutrient transport, concentrate solutes, and cause mechanical damage to cellular structures through ice crystal growth [2,3]. Consequently, microorganisms inhabiting polar environments have evolved a diverse array of cold-adaptation strategies, including membrane remodeling, cryoprotectant production, cold-shock proteins, and antifreeze proteins (AFPs), also known as ice-binding proteins (IBPs) [4,5,6,7].

AFPs and IBPs are specialised proteins that interact directly with ice crystals, thereby modifying ice growth and recrystallisation [8]. These proteins typically function by adsorbing onto ice crystal surfaces, restricting crystal expansion and reducing ice-induced cellular damage [9]. Their activities are commonly manifested as thermal hysteresis and ice recrystallisation inhibition (IRI), both of which contribute to enhanced survival under freezing conditions [10,11]. Although AFPs have been extensively studied in fish, insects, plants, fungi, and several bacterial species, relatively few have been characterised from Antarctic bacteria [10,12,13,14]. Furthermore, the structural diversity and physiological roles of bacterial AFPs remain incompletely understood compared with their eukaryotic counterparts [10].

Members of the genus *Flavobacterium* are widespread in marine, freshwater, glacier, and polar ecosystems and are recognised for their remarkable capacity to thrive under low-temperature conditions [15]. Several Antarctic *Flavobacterium* species have been reported to possess diverse metabolic and stress-response capabilities that facilitate survival in cold environments [16,17,18]. Nevertheless, despite their ecological importance and abundance in polar habitats, the molecular mechanisms underlying subzero adaptation in *Flavobacterium* remain poorly understood [19]. In particular, little is known about the proteins that enable these bacteria to withstand repeated freezing and thawing events that frequently occur in Antarctic environments.

Recent advances in transcriptomics have revealed that a substantial proportion of genes induced during cold stress encode hypothetical proteins with unknown functions [20,21,22,23]. Similar observations have been reported in several psychrophilic and psychrotolerant microorganisms, suggesting that many important cold-adaptation mechanisms remain undiscovered [20,22]. Traditionally, the functional characterization of hypothetical proteins has been hindered by their low sequence similarity to proteins with known functions. However, recent breakthroughs in protein structure prediction, particularly the development of AlphaFold, have enabled reliable functional inference based on tertiary structure rather than sequence homology alone [24,25,26]. Structure-guided approaches therefore provide a powerful strategy for uncovering previously unrecognized proteins involved in environmental adaptation.

Despite increasing recognition of the importance of hypothetical proteins in microbial stress responses, few studies have combined transcriptomic discovery, structural prediction, and experimental validation to investigate their roles in cold adaptation. Moreover, no antifreeze-like proteins have been experimentally characterized from Antarctic *Flavobacterium* species using an integrated structure-guided workflow. This represents a significant knowledge gap in our understanding of bacterial adaptation to subzero environments and limits our ability to identify novel cryoprotective mechanisms employed by polar microorganisms.

To address this gap, we investigated a highly upregulated hypothetical protein, PL002-1792, identified from the Antarctic bacterium *Flavobacterium* sp. PL002 during growth under subzero conditions. Based on its strong induction under severe cold stress, predicted β-sheet-rich architecture, and the presence of sequence motifs associated with bacterial ice-binding proteins, we hypothesized that PL002-1792 functions as an antifreeze-like protein that contributes to cellular protection during freezing stress. We further hypothesized that structural features characteristic of ice-binding proteins would be reflected in measurable ice recrystallisation inhibition activity and enhanced tolerance to repeated freeze–thaw cycles.

Accordingly, the objectives of this study were to (i) identify and prioritize a strongly cold-responsive hypothetical protein from Antarctic *Flavobacterium* sp. PL002, (ii) characterize its structural and physicochemical properties using bioinformatic and structural prediction approaches, (iii) express and experimentally validate the folding of the recombinant protein, and (iv) determine its potential antifreeze and cryoprotective functions through ice recrystallisation inhibition and freeze–thaw survival assays. By integrating transcriptomic analysis, structure-guided prediction, and functional validation, this study provides new insights into the roles of previously uncharacterized proteins in microbial adaptation to extreme cold environments.

## 2. Materials and Methods

### 2.1. Bacterial Strain and Identification

*Flavobacterium* sp. PL002 was isolated from the macroalgae *Porphyra* collected from Ardley Island, Fildes Bay, Antarctica (62°12′30.92″ S, 58°56′26.65″ W). Taxonomic identification was performed using *16S* rRNA gene sequencing and phylogenetic analysis. Genome sequencing and annotation revealed a complete genome of 4.47 Mb with a GC content of 33% and 4291 predicted genes.

### 2.2. Identification of a Cold-Responsive Candidate Protein

PL002-1792 was identified from an unpublished comparative transcriptomic analysis of *Flavobacterium* sp. PL002 cultured under different low-temperature conditions. The transcriptomic analysis served solely as a screening approach to identify highly cold-responsive candidate genes for subsequent structural and functional characterization. As the focus of the present study is the characterization of PL002-1792, only the differential expression results relevant to this candidate protein are presented here. The amino acid sequence of PL002-1792 was retrieved from the annotated genome and analysed using ExPASy ProtParam. Protein length, molecular weight, theoretical isoelectric point (pI), instability index, aliphatic index, and grand average hydropathicity (GRAVY) were calculated [27,28]. These parameters were used to assess the physicochemical characteristics and potential stability of the protein.

### 2.3. Signal Peptide and Motif Analysis

Signal peptide prediction was performed using SignalP 6.0 to determine the presence and type of N-terminal secretion signals [29]. Conserved sequence motifs were analysed by manual inspection and sequence alignment with known bacterial ice-binding proteins. Particular attention was given to glycine-rich regions and TXT-like motifs previously associated with ice-binding activity [30].

### 2.4. Homology Search Against Known Proteins

Sequence homology analysis was performed to identify potential similarities between the candidate protein PL002-1792 and previously characterised proteins. BLASTp searches were conducted against the NCBI non-redundant (nr) protein database and the Protein Data Bank (PDB) to identify related sequences and structural homologs [31,32]. In addition, HHpred was used to detect remote homologies and structural relationships using hidden Markov model comparisons, thereby allowing for the identification of potential structural templates even at low sequence identity [33]. Structural similarity searches were further performed using Foldseek against the PDB database to compare the predicted three-dimensional structure with experimentally determined protein structures [34]. These analyses were carried out to assess the degree of similarity between PL002-1792 and known proteins and to determine whether the protein represents a structurally distinct antifreeze-like protein with low sequence similarity to currently available PDB structures.

### 2.5. Protein Phylogenetic Analysis

To investigate the evolutionary relationship of PL002-1792 with previously reported bacterial ice-binding proteins (IBPs), representative bacterial IBPs and closely related β-helix domain-containing proteins from *Flavobacterium* species were retrieved from the NCBI protein database based on BLASTP analysis. Multiple sequence alignment of the amino acid sequences was performed using ClustalW with default parameters. A phylogenetic tree was reconstructed using the Maximum Likelihood (ML) method implemented in PhyML, and 100 bootstrap replicates evaluated the reliability of the inferred tree topology. The resulting phylogenetic tree was visualized and edited for publication. Branch lengths represent the estimated number of amino acid substitutions per site.

### 2.6. Structural Prediction

The three-dimensional structure of the candidate protein PL002-1792 was predicted using AlphaFold3 (https://alphafoldserver.com/) [24]. AlphaFold3 was selected because it provides improved prediction accuracy for protein tertiary structures, including enhanced backbone geometry, side-chain positioning, and confidence estimation, making it particularly suitable for structural characterisation of previously unannotated proteins lacking experimentally determined homologues. The resulting structural model was evaluated based on the predicted local distance difference test (pLDDT) score, which provides residue-level confidence in the predicted structure, and the predicted template modeling score (pTM), which estimates the overall accuracy of the predicted fold. Residues with pLDDT values above 70 were considered to have reliable structural predictions. The predicted protein structure was subsequently visualised and analysed using ChimeraX to examine the overall fold, secondary structure organisation, and surface features potentially associated with antifreeze-like activity [35].

### 2.7. Structure Validation

The quality and reliability of the predicted three-dimensional structure of PL002-1792 were evaluated using multiple structural validation tools. PROCHECK was used to assess stereochemical quality by generating Ramachandran plots and determining the distribution of residues in favoured, allowed, and disallowed regions [36]. MolProbity was employed to evaluate overall model geometry, including side-chain conformations and steric clashes [37]. In addition, Verify3D was used to assess the compatibility between the predicted three-dimensional structure and its amino acid sequence by evaluating the environmental profile of each residue [38]. Ramachandran plot statistics and other structural quality metrics were used to determine the reliability and overall structural integrity of the predicted protein model.

### 2.8. Structural Comparison with Known Antifreeze Protein

Structural comparison was performed to evaluate the similarity between the predicted structure of PL002-1792 and previously characterised bacterial antifreeze protein. The predicted model was compared with representative bacterial ice-binding proteins (IBPs), including the well-characterised *F. frigoris* ice-binding protein (*FfIBP*) [30,39,40]. The predicted structure of PL002-1792 was compared with the experimentally determined ice-binding protein from *F. frigoris* (*FfIBP*; PDB ID: 4NU2), which served as the reference structure. Structural alignment was performed using TM-align, and structural similarity was assessed based on the root-mean-square deviation (RMSD), template modelling score (TM-score), number of aligned residues, and sequence identity. These quantitative structural metrics were used to evaluate the degree of structural conservation between PL002-1792 and *FfIBP* despite their low amino acid sequence identity. Structural superposition and visualisation were performed using UCSF Chimera. Structure alignments were performed using TM align and structural similarity searches were conducted using the DALI server [41].

### 2.9. Multiple Sequence Alignment and Identification of Conserved Ice-Binding Motifs

Multiple sequence alignment was performed to identify conserved amino acid residues and sequence motifs associated with bacterial ice-binding proteins (IBPs). The amino acid sequence of PL002-1792 was aligned with representative bacterial IBPs and closely related β-helix domain-containing proteins using ClustalW with default parameters. The resulting alignment was visualised using the ESPript 3.x server to identify conserved residues, glycine-rich regions, and repetitive TXT-like motifs that have previously been implicated in ice-binding activity [30,39,40,42,43]. The conservation and spatial distribution of these motifs were subsequently examined in the AlphaFold3-predicted structure of PL002-1792 using UCSF Chimera. Putative ice-binding surfaces were inferred by integrating sequence conservation with structural features, including relatively flat β-sheet surfaces and the arrangement of conserved TXT-like motifs, through comparison with the experimentally characterised ice-binding protein from *F. frigoris* (*FfIBP*; PDB ID: 4NU2).

### 2.10. Recombinant Expression

The gene encoding PL002-1792 was synthesised and cloned into the pET-28a(+) expression vector to enable recombinant protein production with an N-terminal 6×His-tag for purification. The recombinant plasmid was transformed into *E. coli* BL21(DE3) competent cells for protein expression. Expression conditions were optimised by varying induction temperature (8, 10, 12, 20, and 25 °C), IPTG concentration (0.01–0.5 mM), induction duration (16–48 h), and induction optical density (OD_600_ 0.5–0.6). Protein expression and solubility were evaluated by SDS-PAGE analysis of soluble and insoluble fractions following cell lysis. These conditions were selected to enhance soluble expression and minimise inclusion body formation during recombinant protein production.

### 2.11. Protein Refolding and Structural Validation

PL002-1792 was predominantly recovered in the insoluble fraction, where the inclusion bodies were isolated by centrifugation and washed with buffer containing 0.5% Triton X-100 to remove contaminating proteins and membrane components. Purified inclusion bodies were solubilised in 8 M urea containing dithiothreitol (DTT) and incubated at 37 °C for 1 h. Insoluble debris was removed by centrifugation. Protein refolding was achieved by stepwise dialysis at 4 °C over six days with gradual reduction in denaturant concentration and daily buffer replacement. Refolding was performed under continuous stirring to minimise aggregation.

### 2.12. SDS-PAGE and Circular Dichroism Spectroscopy

Refolded PL002-1792 was analysed by SDS-PAGE under reducing and non-reducing conditions to assess protein integrity and aggregation state. Secondary structure content was evaluated using far-UV circular dichroism (CD) spectroscopy. Spectra were recorded between 200 and 290 nm and used to assess the overall folding state of the recombinant protein [44]. The resulting spectra were compared with structural predictions generated by AlphaFold3. Secondary-structure composition was estimated from the far-UV CD spectrum using the BeStSel server, which deconvolutes CD spectra to quantify the relative proportions of α-helices, β-sheets, turns, and other structural elements.

### 2.13. Ice Recrystallisation Inhibition Assay

Ice recrystallisation inhibition (IRI) activity was assessed using a modified splat-cooling assay based on a previously described method [45]. Protein samples (1, 5, and 10 mg mL^−1^) were prepared in Tris-HCl buffer. A 10 μL droplet was released from a height of approximately 1.2 m onto a liquid nitrogen-cooled glass coverslip to generate a thin ice water. The frozen sample was transferred to a Linkam TMHS6000 cold stage (Linkam Scientific Instruments) and annealed at −6 °C for 30 min. Ice crystals were visualized under polarized light microscopy using an Olympus BX51 microscope, and the resulting images were captured with an Olympus DP71 CCD camera. Bovine serum albumin (BSA) 10 mg mL^−1^ was used as a negative protein control. Mean grain size (MGS) was quantified using image analysis software and normalised against the BSA control. Differences in relative mean grain size between the BSA control and PL002-1792 were analysed using an unpaired two-tailed Student’s *t*-test.

### 2.14. Freeze–Thaw Protection Assay

The freeze–thaw assay was adapted from [42] with modifications. The protective effect of PL002-1792 was evaluated using recombinant *E. coli* expressing the target protein. Cultures harboring pET28a-PL002-1792 and the empty pET28a vector were induced and cultivated at either 12 °C or 20 °C for 24 h. Following induction, cells were harvested, washed, and resuspended in sterile 1× phosphate-buffered saline (PBS). The cell density of each culture was adjusted to an OD_600_ of 1.0, corresponding to approximately 5 × 10^8^ CFU mL^−1^ based on a previously established calibration curve. The standardized cell suspensions were subjected to three freeze–thaw cycles. Each cycle consisted of freezing the samples at −80 °C without cryoprotectant for 24 h, followed by thawing at room temperature for 30 min. After each thawing step, serial ten-fold dilutions were prepared in sterile PBS, and 100 μL aliquots were spread onto Luria–Bertani (LB) agar plates containing kanamycin (50 μg mL^−1^). Plates were incubated at 37 °C for 24 h, after which colony-forming units (CFUs) were enumerated. Cell survival was expressed as the percentage of viable cells remaining after each freeze–thaw cycle relative to the initial CFU count before freezing, calculated as Survival (%) = (CFUs after freeze thaw/Initial CFUs) × 100. All experiments were performed using three independent biological replicates. Data are presented as the mean ± standard deviation. Statistical significance was determined using two-way analysis of variance (ANOVA) followed by Tukey’s multiple comparison test, with differences considered statistically significant at *p* < 0.05.

## 3. Results

### 3.1. Genome Features and Identification of a Cold-Responsive Candidate Protein

Phylogenetic analysis based on the *16S* rRNA gene confirmed that strain PL002 belongs to the genus *Flavobacterium* [46]. The isolate clustered within the *Flavobacterium* clade and showed close phylogenetic relationships with several cold-adapted *Flavobacterium* species, including *F. frigoris* and *F. frigidarium*, supporting its taxonomic placement within this genus. These findings are consistent with the ecological origin of PL002 from Antarctic marine environments, where members of the genus are commonly associated with cold-adapted microbial communities.

Genome sequencing and annotation revealed that *Flavobacterium* sp. PL002 possesses a complete genome of approximately 4.47 Mb, with a GC content of 33%. A total of 4291 protein-coding sequences (CDSs) were predicted, encompassing genes associated with central metabolism, environmental sensing, nutrient acquisition, and stress adaptation. Notably, a substantial proportion of the predicted CDSs were annotated as hypothetical proteins, indicating the presence of numerous uncharacterized genes that may contribute to adaptation in extreme Antarctic environments [46]. An unpublished comparative transcriptomic analysis performed under different low-temperature conditions identified PL002-1792 as one of the most strongly induced hypothetical proteins. Because the objective of the present study was to characterize the biological function of PL002-1792, only the differential expression data relevant to this candidate protein are presented. Comparative transcriptomic analysis included two temperature comparisons: −20 °C versus 15 °C and −20 °C versus −6 °C. As 15 °C represents the optimal growth temperature of *Flavobacterium* sp. PL002, the former comparison was used to identify genes involved in the general cold response, whereas the latter was emphasised because it identifies genes specifically associated with adaptation to extreme subzero conditions. PL002-1792 was significantly upregulated in both comparisons, exhibiting a log_2_ fold change of 5.61 (adjusted *p* = 1.89 × 10^−138^) for −20 °C versus −6 °C and 4.89 (adjusted *p* = 1.10 × 10^−59^) for −20 °C versus 15 °C (Table 1).

Despite its strong induction under cold stress, PL002-1792 was annotated as a hypothetical protein with no experimentally validated function. Domain analysis identified the presence of an LIPL48-related domain, while BLASTp searches revealed similarity to hypothetical proteins from other *Flavobacterium* species, suggesting that the protein belongs to a poorly characterised family of bacterial proteins. Given its exceptionally high level of induction under subzero conditions and lack of functional annotation, PL002-1792 was selected for further structural and functional characterisation as a potential contributor to cold adaptation in Antarctic *Flavobacterium* sp. PL002.

### 3.2. Sequence Analysis and Physicochemical Properties of PL002-1792

The PL002-1792 gene encodes a protein consisting of 402 amino acid residues with a predicted molecular weight of 41.3 kDa, which is comparable to the size of several bacterial β-helix ice-binding proteins. Physicochemical analysis using the ExPASy ProtParam server predicted a theoretical isoelectric point (pI) of 4.70, indicating that the protein is acidic in nature [27]. Acidic proteins are frequently reported in psychrophilic microorganisms, where increased surface acidity enhances protein hydration and conformational flexibility at low temperatures. The instability index was calculated to be 12.66, classifying the protein as stable under physiological conditions. In addition, the aliphatic index of 75.02 suggests moderate structural stability, while the GRAVY value of −0.044 indicates that the protein is slightly hydrophilic. The calculated physicochemical parameters indicate that PL002-1792 is a moderately sized acidic protein with predicted stability and a slightly hydrophilic character. This relatively balanced hydrophobicity is consistent with proteins that interact with aqueous environments and is characteristic of many extracellular or cell surface-associated bacterial ice-binding proteins. A summary of the physicochemical properties of PL002-1792 is presented in Table 2.

### 3.3. Signal Peptide Prediction and Conserved Motif Analysis of PL002-1792

Signal peptide analysis using SignalP 6.0 predicted that PL002-1792 contains an N-terminal lipoprotein signal peptide (Table 3). The protein was classified as a Sec/SPII substrate with a prediction probability of 0.997. The most probable signal peptide cleavage site was identified between amino acid residues 21 and 22, indicating that the mature protein is generated following removal of the N-terminal signal peptide. The presence of a Sec/SPII signal peptide predicts that PL002-1792 is processed through the bacterial Sec secretion pathway and may become associated with the cell envelope following lipid modification. No alternative signal peptide types, including Sec/SPI, Tat/SPI, Tat/SPII, or Sec/SPIII pathways, were predicted. Sequence analysis further identified several glycine-rich regions and multiple TXT-like motifs distributed throughout the protein sequence. These motifs have previously been reported in bacterial ice-binding proteins and were therefore selected for further structural analysis. The predicted signal peptide and conserved sequence motifs indicate that PL002-1792 possesses sequence features commonly associated with extracellular or surface-associated proteins. To further investigate whether these conserved sequence features are associated with ice-binding function, the three-dimensional structure of PL002-1792 was predicted and analyzed using AlphaFold3.

### 3.4. Protein Phylogenetic Analysis

**Figure 1:**
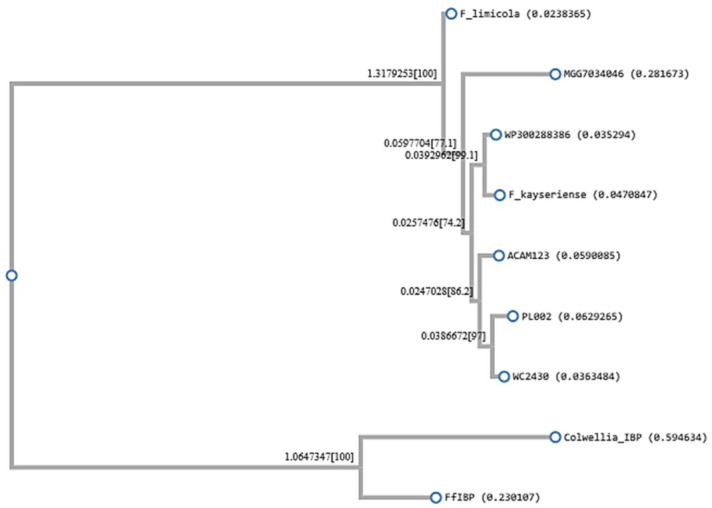
Maximum Likelihood phylogenetic tree of PL002-1792 and representative bacterial ice-binding proteins. The phylogenetic tree was reconstructed from amino acid sequences aligned using ClustalW and inferred using the Maximum Likelihood method implemented in PhyML. Bootstrap values (100 replicates) are shown at the nodes, and branch lengths represent the estimated number of amino acid substitutions per site.

A Maximum Likelihood phylogenetic analysis was performed to determine the evolutionary relationship of PL002-1792 with representative bacterial ice-binding proteins and homologous β-helix domain-containing proteins (Figure 1). The phylogenetic tree resolved two major lineages. One lineage comprised the experimentally characterized ice-binding proteins from *F. frigoris* (*FfIBP*) and *Colwellia* sp., whereas the second lineage consisted of β-helix domain-containing proteins from *Flavobacterium* species, including PL002-1792. Within the *Flavobacterium* lineage, PL002-1792 formed a strongly supported sister relationship with the homolog from *Flavobacterium* sp. WC2430 (bootstrap = 97%). This subgroup clustered with the protein from *Flavobacterium* sp. ACAM123 (bootstrap = 86.2%), while the homologs from *Flavobacterium kayseriense* and *Flavobacterium* sp. WP300288386 formed another well-supported clade (bootstrap = 99.1%). *Flavobacterium limicola* and the hypothetical protein from *Flavobacterium* sp. MGG7034046 occupied more basal positions within the *Flavobacterium* β-helical IBP lineage. Overall, the phylogenetic analysis supports the close evolutionary relationship of PL002-1792 with β-helical ice-binding proteins from *Flavobacterium* species.

### 3.5. Structural Prediction and Comparative Analysis of PL002-1792

The three-dimensional structure of PL002-1792 was predicted using AlphaFold3 to gain insight into its potential function. The resulting model exhibited a high predicted template modeling (pTM) score of 0.96, indicating a highly reliable overall fold prediction [47] (Figure 1).

To further evaluate the reliability of the AlphaFold3-predicted structure, the model was assessed using standard stereochemical and structural validation tools (Table 4). The model exhibited 95.38% of residues in the favoured Ramachandran regions and 4.62% in additionally allowed regions, with no Ramachandran or rotamer outliers. The model also achieved a MolProbity score of 1.85, a clashscore of 8.59, a Verify3D score of 91.98%, and an ERRAT overall quality factor of 90.33%, indicating excellent stereochemical quality and structural reliability. Together with the high AlphaFold3 pTM score of 0.96, these validation metrics demonstrate that the predicted model provides a robust structural framework for subsequent comparative and functional analyses.

**Figure 2:**
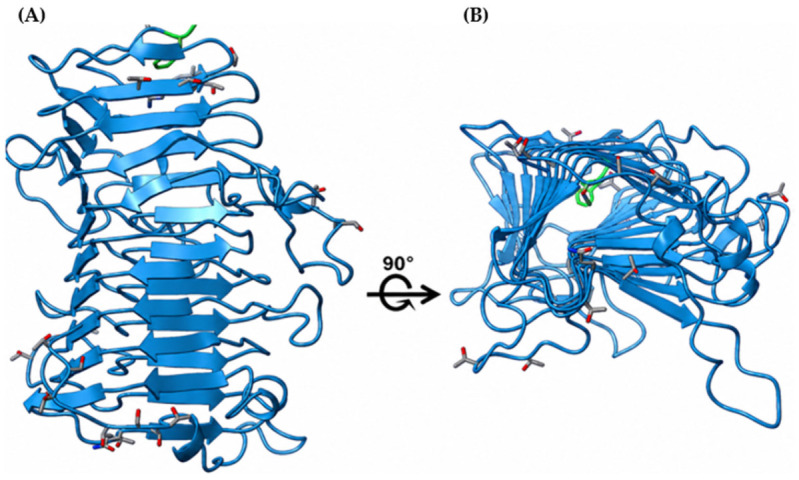
AlphaFold3-predicted structure of PL002-1792 shown in blue. (**A**) Ribbon representation showing the overall fold of the protein. (**B**) Structure rotated by 90° to illustrate the elongated β-sheet-rich architecture. The predicted model exhibited a high-confidence pTM score of 0.96 and was dominated by stacked β-strands interconnected by short loop regions.

**Figure 3:**
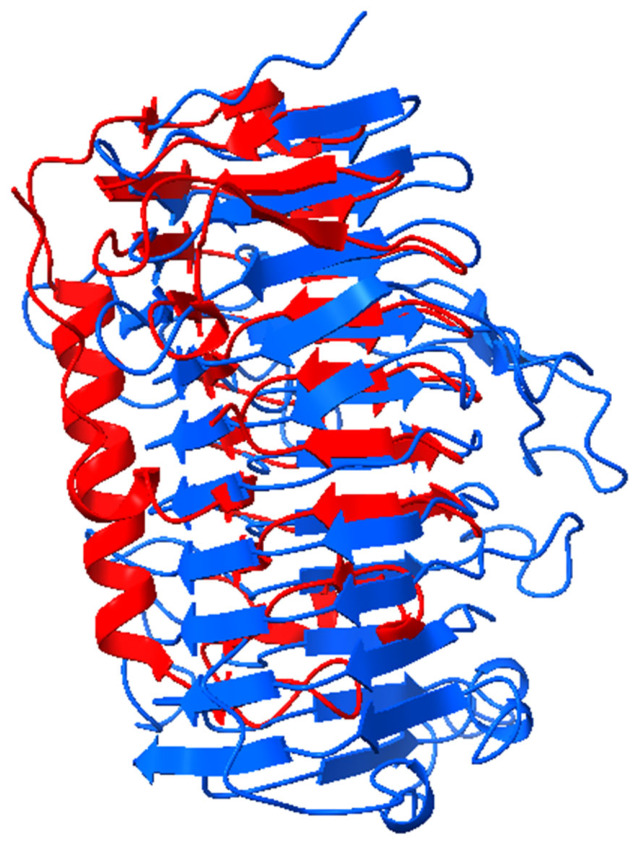
Structural superposition of the AlphaFold3-predicted structure of PL002-1792 (blue) and the experimentally determined ice-binding protein from *F. frigoris* (*FfIBP*; PDB ID: 4NU2) (red) generated using UCSF Chimera. The superimposed structures reveal conservation of the characteristic β-helical scaffold despite low amino acid sequence identity. Structural alignment of the conserved core yielded an RMSD of 1.25 Å (26 pruned atom pairs), indicating a high degree of local structural similarity, whereas TM-align analysis of the full-length structures aligned 122 residues with an RMSD of 3.78 Å and a TM-score of 0.414, supporting overall structural conservation despite sequence divergence.

Visualization of the predicted structure revealed an elongated architecture dominated by β-strands interconnected by short loop regions (Figure 2A). The protein adopted a β-sheet-rich fold extending throughout the length of the molecule, producing an overall topology characteristic of several previously described bacterial ice-binding proteins. Examination of the structure from different orientations demonstrated the presence of stacked β-sheet surfaces forming an elongated β-solenoid-like scaffold (Figure 2B). The N- and C-terminal regions were positioned at opposite ends of the molecule, resulting in an extended structure with relatively flat surface regions. The predominance of β-sheet secondary structure observed in the predicted model was consistent with the sequence-derived characteristics of PL002-1792 and was subsequently evaluated experimentally using circular dichroism spectroscopy. Quantitative structural comparison was performed using TM-align against the experimentally determined structure of the *F. frigoris* ice-binding protein (PDB ID: 4NU2). The structural alignment identified 122 aligned residues with an RMSD of 3.78 Å, a TM-score of 0.414 (normalised to the reference structure), and 9.8% sequence identity. Although the amino acid sequence identity was low, the structural alignment demonstrated conservation of the β-helical scaffold characteristic of bacterial ice-binding proteins. Structural superposition using UCSF Chimera further demonstrated that the conserved structural core of the two proteins could be superimposed with an RMSD of 1.25 Å after iterative pruning of poorly aligned regions (26 atom pairs), indicating a high degree of local structural similarity within the conserved β-helical core (Figure 3). The β-solenoid core was well conserved between the two structures, whereas structural differences were primarily observed in the terminal regions and surface-exposed loops. Multiple conserved TXT motifs were distributed along the flat β-sheet surface of PL002-1792 in positions corresponding to putative ice-binding regions previously reported in *FfIBP*, suggesting conservation of structural features associated with ice-binding function.

**Figure 4:**
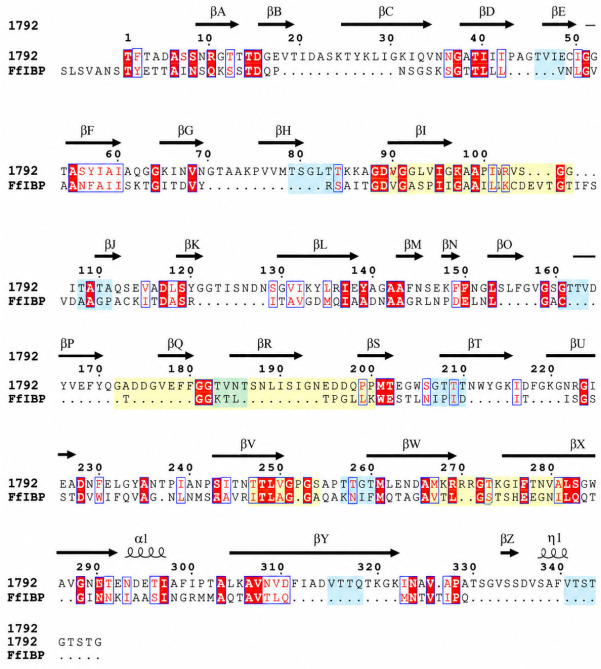
Sequence alignment of PL002-1792 with the experimentally characterised ice-binding protein from *F. frigoris* (*FfIBP*). Conserved residues are highlighted in red. Blue and yellow boxes indicate putative TXT-like motifs and glycine-rich regions, respectively. Predicted secondary structure elements are shown above the alignment, with β-strands (βA–βZ) represented by arrows and α-helices (α1) by coils. Despite low overall sequence identity, PL002-1792 retains structural features characteristic of bacterial ice-binding and antifreeze-like proteins, including TXT-like motifs, glycine-rich regions, and a β-strand-rich architecture.

To investigate structural similarity with known bacterial ice-binding proteins, PL002-1792 was compared with the ice-binding protein from *F. frigoris* (*FfIBP*). Multiple sequence alignment revealed several conserved residues located within predicted β-strand regions despite relatively low overall sequence identity (Figure 4).

In addition, multiple glycine-rich segments and TXT-like motifs were identified throughout the sequence. These motifs were distributed within regions corresponding to the predicted β-sheet-rich scaffold and were retained in both PL002-1792 and *FfIBP*. Comparison of the aligned sequences further showed conservation of several residues associated with repetitive β-strand architecture characteristic of bacterial ice-binding proteins. Although extensive sequence divergence was observed across much of the protein sequence, the overall arrangement of conserved motifs and β-strand-associated residues remained evident. The preservation of these sequence features, together with the highly similar β-sheet-rich structural organisation predicted by AlphaFold3, indicated structural conservation between PL002-1792 and previously characterised bacterial ice-binding proteins. Collectively, the structural prediction and comparative sequence analyses demonstrated that PL002-1792 possesses an elongated β-sheet-rich architecture, conserved glycine-rich regions, and multiple TXT-like motifs commonly observed in bacterial ice-binding proteins. These features provided the basis for subsequent experimental characterisation of the protein.

### 3.6. Recombinant Expression of PL002-1792

**Figure 5:**
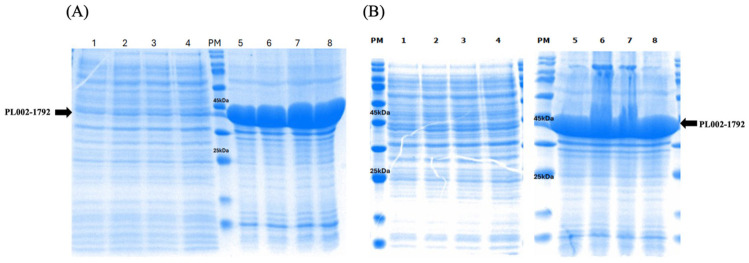
Optimisation of recombinant expression of PL002-1792 in *E. coli* BL21(DE3). SDS-PAGE analysis of recombinant PL002-1792 expression following IPTG induction under different conditions. (**A**) Induction with 0.5 mM IPTG at 20 °C for 16 h; (**B**) induction with 0.5 mM IPTG at 20 °C for 48 h. For each condition, total cell lysate, soluble fraction (supernatant), and insoluble fraction (pellet) were analysed to evaluate protein expression and solubility. Lanes 1–4 correspond to soluble fractions, whereas lanes 5–8 correspond to insoluble pellet fractions. The expected molecular mass of PL002-1792 is approximately 41 kDa. PM, broad-range protein molecular weight marker (SMOBIO, Taiwan).

**Figure A1:**
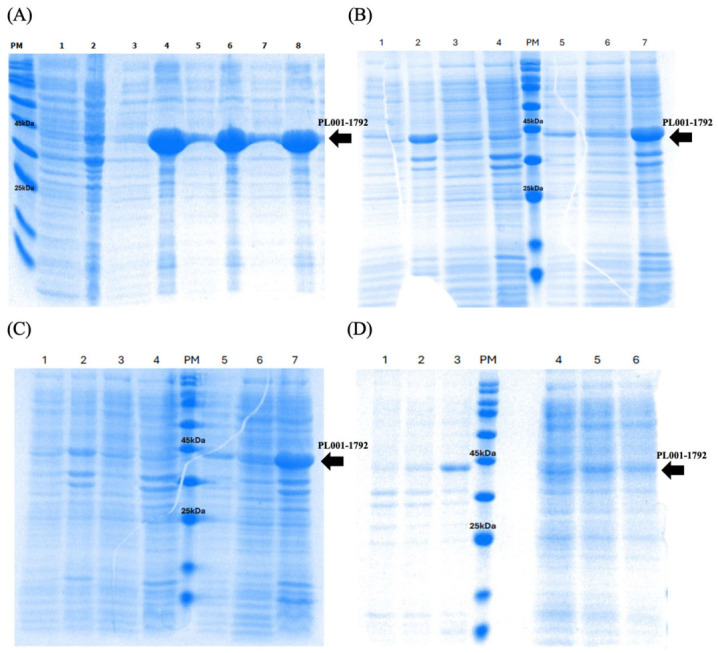
Optimisation of recombinant PL002-1792 expression in *E. coli* BL21(DE3) under different induction conditions. SDS-PAGE analysis of recombinant PL002-1792 expression and solubility following IPTG induction. (**A**) Induction at 25 °C for 16 h. Lane 1, uninduced supernatant; lane 2, induced supernatant; lanes 3, 5, and 7, induced supernatant fractions following induction with 0.01, 0.05, and 0.1 mM IPTG, respectively; lanes 4, 6, and 8, corresponding pellet fractions following induction with 0.01, 0.05, and 0.1 mM IPTG, respectively. (**B**) Induction with 0.01 mM IPTG at 8 °C for 48 h. Lane 1, uninduced supernatant; lane 2, uninduced pellet; lane 3, induced empty-vector control (supernatant); lane 4, induced empty-vector control (pellet); PM, protein molecular weight marker (SMOBIO, Taiwan); lane 5, whole-cell lysate; lane 6, induced PL002-1792 (supernatant); lane 7, induced PL002-1792 (pellet). (**C**) Induction with 0.01 mM IPTG at 10 °C for 48 h. Lane 1, uninduced supernatant; lane 2, uninduced pellet; lane 3, induced empty-vector control (supernatant); lane 4, induced empty-vector control (pellet); PM, protein molecular weight marker (SMOBIO, Taiwan); lane 5, whole-cell lysate; lane 6, induced PL002-1792 (supernatant); lane 7, induced PL002-1792 (pellet). (**D**) Induction at 12 °C for 48 h using IPTG concentrations ranging from 0.01 to 0.1 mM. Lanes 1–3, pellet fractions following induction with 0.01, 0.05, and 0.1 mM IPTG, respectively; lanes 4–6, corresponding supernatant fractions following induction with 0.01, 0.05, and 0.1 mM IPTG, respectively. A prominent protein band corresponding to the predicted molecular mass of PL002-1792 (~41 kDa) was detected under all induction conditions and was predominantly recovered in the insoluble pellet fraction. Arrows indicate the position of recombinant PL002-1792.

Recombinant expression of PL002-1792 was evaluated under multiple induction conditions to optimise protein production and solubility. Expression was performed in *E. coli* BL21(DE3) using a pET-28a(+) expression system, with induction temperatures ranging from 8 to 25 °C, induction periods of 16–48 h, and IPTG concentrations between 0.01 and 0.5 mM. SDS-PAGE analysis consistently revealed the production of a prominent protein band at approximately 41 kDa, corresponding to the predicted molecular weight of PL002-1792 (Figure 3). Initially, protein expression was tested at 20 °C, induced at 0.5mM IPTG at different OD600, but overexpression was found in pellet fractions (Figure 5A,B). Next, recombinant protein expression was optimised by evaluating different induction temperatures (25, 20, 12, 10, and 8 °C), IPTG concentrations, and induction periods (16–48 h) (Figure A1(A–D)). Recombinant protein expression was successfully detected under all conditions tested, confirming heterologous production of PL002-1792 in *E. coli*. Comparison of soluble and insoluble fractions showed that the majority of the recombinant protein was recovered in the pellet fraction. This pattern was observed at all induction temperatures tested, including 25, 20, 12, 10, and 8 °C. Extending the induction period from 16 h to 48 h at 20 °C did not substantially alter the distribution of the protein between soluble and insoluble fractions. Similarly, reducing the induction temperature and IPTG concentration did not result in a noticeable increase in soluble protein recovery. Across all expression conditions examined, only trace amounts of PL002-1792 were detected in the soluble fraction, whereas a strong protein band corresponding to approximately 41 kDa was consistently observed in the pellet fraction. Although expression was detected under all induction conditions, expression at 20 °C for 24 h using 0.5 mM IPTG was selected for subsequent protein production because it provided consistent protein expression for downstream purification and refolding. These results indicate that recombinant PL002-1792 was predominantly produced as insoluble protein during heterologous expression.

### 3.7. Recovery and Refolding of Recombinant PL002-1792

Since recombinant PL002-1792 was predominantly recovered in the insoluble fraction, inclusion bodies were isolated for subsequent protein recovery. Following cell lysis and centrifugation, the insoluble pellet containing PL002-1792 was collected and washed to remove contaminating cellular proteins and membrane components. SDS-PAGE analysis confirmed enrichment of the target protein in the purified inclusion body fraction. The isolated inclusion bodies were subsequently solubilized in denaturing buffer containing 8 M urea and dithiothreitol (DTT), resulting in complete dissolution of the protein. Insoluble debris was removed by centrifugation, and the denatured protein solution was subjected to stepwise dialysis-based refolding at 4 °C. Refolding was performed over six days with gradual removal of the denaturant through sequential buffer exchanges. The refolding procedure yielded soluble recombinant PL002-1792 suitable for downstream structural and functional analyses. The recovered protein was subsequently evaluated by SDS-PAGE and circular dichroism spectroscopy to assess its structural integrity and folding status.

### 3.8. Structural Validation of Refolded PL002-1792

**Figure 6:**
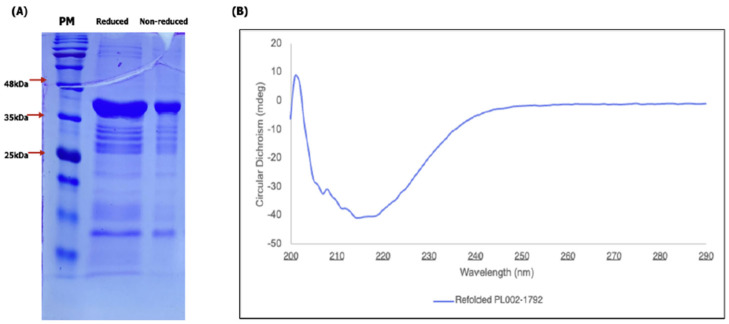
Structural validation of refolded PL002-1792. (**A**) SDS-PAGE analysis of refolded protein under reducing (R) and non-reducing (NR) conditions showing a predominant protein band at approximately 41 kDa. (**B**) Far-UV circular dichroism spectrum of refolded PL002-1792 displaying a pronounced negative ellipticity minimum at 216–218 nm, indicative of a β-sheet-rich secondary structure. The experimental CD profile was consistent with the β-sheet-rich architecture predicted by AlphaFold3.

The structural integrity of refolded PL002-1792 was evaluated using SDS-PAGE and circular dichroism (CD) spectroscopy. SDS-PAGE analysis under both reducing and non-reducing conditions revealed a prominent protein band at approximately 41 kDa, corresponding closely to the predicted molecular weight of PL002-1792 (Figure 6A). Similar migration patterns were observed under both conditions, indicating that the protein remained predominantly monomeric following refolding. No substantial high-molecular-weight species or visible protein aggregates were detected, suggesting successful recovery of a soluble protein suitable for downstream functional analyses. The secondary structure of refolded PL002-1792 was further examined using far-UV CD spectroscopy. The CD spectrum exhibited a pronounced negative ellipticity minimum centred between 216 and 218 nm (Figure 6B), a characteristic feature of β-sheet-rich proteins. Quantitative secondary-structure analysis using the BeStSel server estimated that PL002-1792 contains 38.4% β-sheet structure, comprising 17.9% relaxed antiparallel β-sheet, 19.8% left-twisted antiparallel β-sheet, and 0.7% parallel β-sheet, together with 16.1% turns and only 1.6% α-helical content (Table 5). To quantitatively validate the AlphaFold3 model, the predicted structure was analysed using the DSSP algorithm. DSSP estimated that PL002-1792 contains 47.6% β-sheet, 12.9% turns, 2.0% α-helix, and 37.5% coil/other (Table 4). Although minor differences were observed between the computational and experimental estimates, both analyses consistently indicate that PL002-1792 is a predominantly β-sheet-rich protein with minimal α-helical content, supporting the β-solenoid architecture predicted by AlphaFold3. Collectively, the CD spectroscopy and DSSP analyses provide complementary evidence that the refolded recombinant protein adopts the β-sheet-dominated conformation predicted in silico.

### 3.9. Ice Recrystallisation Inhibition Activity of PL002-1792

**Figure 7:**
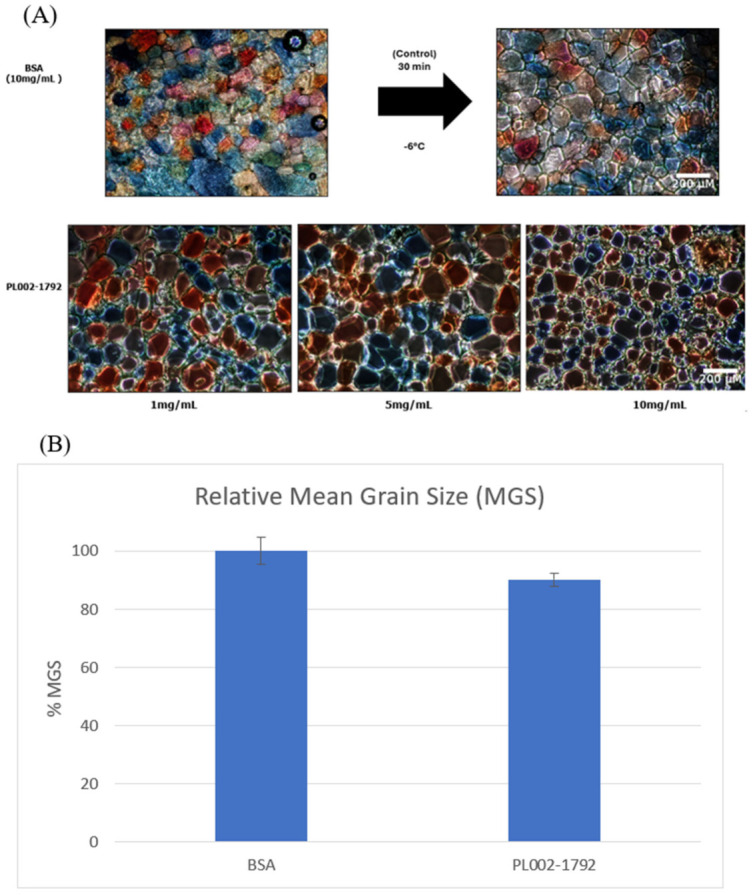
Ice recrystallization inhibition activity of PL002-1792. (**A**) Representative polarized light micrographs of ice crystals following annealing at −6 °C for 30 min in the presence of bovine serum albumin (BSA) or PL002-1792 at different protein concentrations. Samples containing PL002-1792 exhibited smaller ice crystals compared with the BSA control. (**B**) Relative mean grain size (MGS) of ice crystals normalised to the BSA control (10 mg mL^−1^). Greatest inhibition observed at 10 mg mL^−1^. PL002-1792 reduced ice crystal grain size to approximately 90% of the control, indicating moderate ice recrystallisation inhibition activity. Values represent the mean standard deviation (SD) of three independent biological replicates. Statistical significance between the BSA control and PL002-1792 was determined using an unpaired two-tailed Student’s *t*-test (*p* < 0.001).

The ice recrystallisation inhibition (IRI) activity of PL002-1792 was evaluated using a splat-cooling assay followed by annealing at −6 °C for 30 min. Ice crystal morphology was examined under polarised light microscopy and compared with bovine serum albumin (BSA), which served as a negative protein control (Figure 7A). Following annealing, the BSA control exhibited relatively large and well-developed ice crystals characteristic of extensive ice recrystallisation. In contrast, samples containing PL002-1792 displayed visibly smaller ice crystals, particularly at higher protein concentrations. The reduction in ice crystal size was most evident at a protein concentration of 10 mg mL^−1^, where the formation of large recrystallised ice grains was noticeably reduced compared with the control. Quantitative analysis of relative Mean Grain Size (MGS) supported these observations (Figure 7B). When normalised against the BSA control, PL002-1792 reduced ice crystal grain size to approximately 90% of the control value. The reduction in MGS was observed across the tested protein concentrations and was most pronounced at 10 mg mL^−1^. These results demonstrate that PL002-1792 possesses measurable ice recrystallisation inhibition activity and is capable of reducing ice crystal growth during annealing under freezing conditions.

**4.0. PL002-1792 Enhances Freeze–Thaw Tolerance**

**Figure 8:**
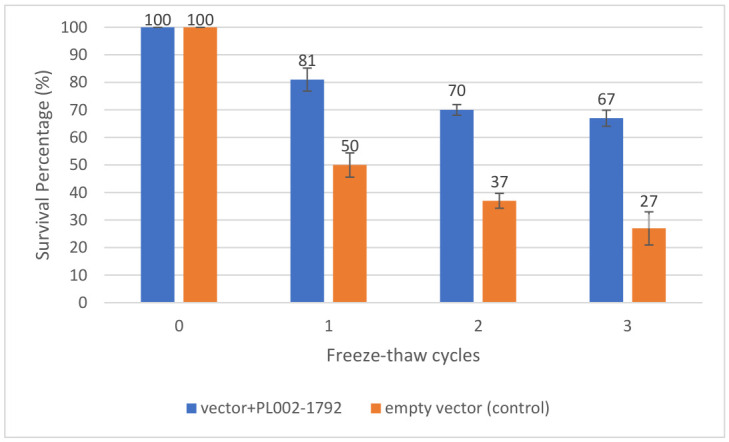
Freeze–thaw survival of recombinant *E. coli* expressing PL002-1792. Cultures harboring pET28a-PL002-1792 (vector_PL002-1792) or the empty pET28a vector were subjected to four successive freeze–thaw cycles. Cell viability was determined by colony-forming unit (CFU) enumeration after each thawing step and expressed as the percentage of surviving cells relative to the initial CFU count before freezing. Values represent the mean ± SD of three independent biological replicates. Statistical significance was determined using two-way ANOVA (*p* < 0.01) followed by Tukey’s multiple comparison test.

To evaluate the protective effect of PL002-1792 against freezing stress, recombinant *E. coli* expressing PL002-1792 and empty-vector control cells were subjected to repeated freeze–thaw cycles. Cell survival was determined after each cycle and expressed as a percentage relative to the initial cell density before freezing (Figure 8). Both strains exhibited a progressive decline in survival with increasing numbers of freeze–thaw cycles. However, cells expressing PL002-1792 consistently maintained significantly higher survival rates than the empty-vector control throughout the experiment. Following the first freeze–thaw cycle, PL002-1792-expressing cells retained 81% viability, whereas the empty-vector control retained only 50%. After the second cycle, viability declined to 70% in PL002-1792-expressing cells, compared with only 37% in the empty-vector control. Following the third freeze–thaw cycle, recombinant cells expressing PL002-1792 maintained 67% viability, while survival of the empty-vector control decreased further to 27%. The enhanced survival observed in cells expressing PL002-1792 demonstrates that the protein confers substantial protection against freeze–thaw-induced cellular damage. The ability of PL002-1792 to maintain cell viability during repeated freezing and thawing is consistent with the proposed function of the protein as a cryoprotective or ice-binding protein and supports its potential role in improving cellular tolerance to freezing stress.

## 4. Discussion

The ability to survive prolonged freezing and repeated freeze–thaw events is a fundamental requirement for microbial persistence in Antarctic environments [48,49,50]. Such conditions expose cells to ice crystal formation, osmotic stress, membrane damage, and macromolecular destabilisation [12]. In the present study, we applied a structure-guided discovery approach to investigate a strongly cold-responsive hypothetical protein, PL002-1792, from Antarctic *Flavobacterium* sp. PL002. By integrating transcriptomic analysis, structural prediction, recombinant expression, and functional assays, we provide evidence that PL002-1792 represents a previously uncharacterized antifreeze-like protein involved in cold adaptation. These species are commonly associated with polar and glacial environments and are known to possess specialised mechanisms for growth at low temperatures. The close phylogenetic relationship suggests that strain PL002 may share similar cold-adaptive traits, providing ecological support for the identification of cold-responsive genes involved in subzero survival. The transcriptomic analysis was used solely as a discovery tool to identify strongly cold-responsive candidate genes. Rather than presenting a comprehensive transcriptomic study, the present work focuses on the structural prediction and experimental validation of PL002-1792. This targeted approach enabled functional characterization of a previously unannotated protein identified through transcriptomic screening. Transcriptomic screening identified PL002-1792 as one of the most strongly induced genes under severe cold stress (Table 1). The observed log_2_ fold changes of 5.61 and 4.89 under −20 °C conditions indicate that expression of PL002-1792 is highly responsive to freezing temperatures. Such dramatic transcriptional induction is consistent with genes that perform important physiological functions during environmental stress. Interestingly, PL002-1792 was annotated only as a hypothetical protein despite its strong regulation. Similar observations have been reported in psychrophilic microorganisms, where many of the most highly induced cold-responsive genes lack functional annotation [20]. This highlights the limitations of sequence-based annotation and emphasises the importance of combining transcriptomics with structural prediction to uncover previously unrecognised stress-response proteins.

The physicochemical properties of PL002-1792 (Table 2) further support a role in cold adaptation. The protein possesses a relatively low theoretical pI (4.70), indicating an acidic character. Acidic proteins are frequently enriched in psychrophilic organisms because increased surface charge can enhance hydration and maintain conformational flexibility at low temperatures [51,52,53]. In addition, the low instability index suggests that PL002-1792 is intrinsically stable despite its predicted flexibility. Furthermore, the aliphatic index of 75.02 suggests moderate structural stability while maintaining sufficient flexibility, a characteristic frequently associated with proteins adapted to cold environments. The slightly negative GRAVY value indicates a predominantly hydrophilic nature, a characteristic commonly observed among extracellular or surface-associated proteins that interact with aqueous environments [28]. Together, these properties are consistent with proteins adapted for function under cold and ice-associated conditions. Signal peptide analysis revealed that PL002-1792 contains a Sec/SPII lipoprotein signal peptide with a highly confident prediction score (Table 3) [29]. This finding suggests that the mature protein is exported through the Sec secretion pathway and subsequently anchored to the cell envelope through lipid modification [54]. Many bacterial ice-binding proteins are secreted or cell-surface associated, enabling them to interact directly with extracellular ice crystals [12,30,47]. Notably, the ice-binding protein from *F. frigoris* (*FfIBP*) has been shown to function as a membrane-anchored protein that promotes the formation of unfrozen brine pockets around the cell during freezing, thereby reducing local salt concentration and enhancing microbial survival under freezing conditions [35]. Furthermore, the structural rigidity of *FfIBP* is critical for its hyperthermal hysteresis activity and its ability to enhance microbial survival during freeze–thaw stress, highlighting the importance of maintaining the structural integrity of bacterial ice-binding proteins [42]. Given that PL002-1792 also possesses a Sec/SPII lipoprotein signal peptide and adopts a conserved β-solenoid architecture, it is plausible that it employs a similar membrane-associated strategy to facilitate interactions with extracellular ice and improve freeze tolerance. Nevertheless, the cellular localisation and physiological role of PL002-1792 remain to be experimentally confirmed [42]. The subcellular localisation of PL002-1792 was inferred solely from bioinformatic prediction and was not experimentally confirmed in the present study. Therefore, although the predicted localisation is consistent with its proposed antifreeze-like function, experimental validation through subcellular fractionation, immunolocalization, or fluorescence-based localisation studies will be necessary to confirm its native cellular localisation in *Flavobacterium* sp. PL002.

The phylogenetic analysis provides additional evidence supporting the classification of PL002-1792 as a member of the bacterial β-helical ice-binding protein family (Figure 1). PL002-1792 clustered most closely with the homolog from *Flavobacterium* sp. WC2430, with strong bootstrap support (97%), indicating that these proteins likely share a recent common ancestor. The close association with other *Flavobacterium* homologs suggests that PL002-1792 belongs to a conserved lineage of β-helical ice-binding proteins that has diversified within cold-adapted *Flavobacterium* species. In contrast, the experimentally characterized *FfIBP* and the *Colwellia* ice-binding protein formed a distinct lineage, indicating greater evolutionary divergence while retaining the conserved functional role of ice binding. These phylogenetic findings are consistent with the sequence conservation, predicted β-helical structure, and experimentally demonstrated ice recrystallisation inhibition activity of PL002-1792.

One of the most striking findings of this study was the structural similarity between PL002-1792 and known bacterial ice-binding proteins. AlphaFold3 prediction generated a highly confident model with a pTM score of 0.96, indicating strong reliability of the predicted fold (Figure 2) [24,47]. The model revealed an elongated architecture dominated by β-sheets arranged into a β-solenoid-like scaffold. This structural organisation closely resembles that reported for several bacterial ice-binding proteins, including the well-characterised ice-binding protein from *F. frigoris* (*FfIBP*) [30,39,40]. Quantitative structural comparison further supported this observation (Figure 3). TM-align analysis of PL002-1792 and *FfIBP* yielded a TM-score of 0.414 despite only 9.8% sequence identity, indicating meaningful conservation of the overall structural framework. Furthermore, structural superposition using UCSF Chimera demonstrated a conserved β-helical core with an RMSD of 1.25 Å after iterative pruning of poorly aligned regions. Together, these findings illustrate that protein tertiary structure can remain conserved despite substantial sequence divergence, supporting the concept that structural conservation is often more strongly associated with protein function than primary sequence similarity [55]. Such observations highlight the value of modern structure prediction tools for identifying proteins that would otherwise remain functionally uncharacterized [26].

Despite the relatively low overall sequence identity, PL002-1792 retains several conserved residues, glycine-rich regions, and putative TXT-like motifs that have previously been associated with bacterial ice-binding proteins (Figure 4) [30,39]. The sequence comparison with *FfIBP* provided additional evidence supporting an antifreeze-like function. Multiple glycine-rich regions and TXT-like motifs were identified within PL002-1792. Similar motifs have previously been associated with ice-binding surfaces in bacterial antifreeze-like proteins, where regularly spaced threonine residues facilitate ordered interactions with water molecules and ice lattices [39,40,56]. Although extensive sequence divergence was observed between PL002-1792 and *FfIBP*, the conservation of motif organisation and β-strand architecture suggests evolutionary preservation of structural features important for ice interaction [30,55]. The combination of motif conservation and structural similarity strongly supports the hypothesis that PL002-1792 belongs to the broader family of bacterial ice-binding proteins [12]. However, the proposed ice-binding surface is inferred from sequence conservation and structural prediction and has not been experimentally validated. Therefore, site-directed mutagenesis of the putative TXT-like motifs, followed by functional analysis, will be required to confirm their contribution to ice-binding activity.

Recombinant expression studies revealed that PL002-1792 was produced efficiently in *E. coli* but accumulated predominantly as inclusion bodies regardless of induction temperature, IPTG concentration, or induction duration (Figure 5). This behaviour is not uncommon among β-sheet-rich proteins and has been reported for several recombinant antifreeze-like proteins expressed in heterologous hosts [57]. The extensive β-sheet architecture predicted for PL002-1792 may promote intermolecular interactions during overexpression, leading to aggregation and inclusion body formation [58]. While insolubility complicated protein recovery, successful refolding from inclusion bodies enabled subsequent structural and functional characterisation [57]. The ability to recover soluble protein after denaturation also suggests that the protein possesses an intrinsically stable fold capable of re-establishing its native conformation following refolding.

Experimental validation of the refolded protein provided strong support for the AlphaFold3 structural prediction. SDS-PAGE analysis confirmed recovery of a monomeric protein with an expected molecular mass of approximately 41 kDa, while circular dichroism (CD) spectroscopy revealed a pronounced minimum at 216–218 nm characteristic of β-sheet-rich proteins (Figure 6) [44]. These experimental findings were in good agreement with the secondary-structure composition predicted from the AlphaFold3 model using the DSSP algorithm, which likewise indicated a predominantly β-sheet-rich protein with minimal α-helical content. Together, these independent computational and experimental analyses support the proposed β-solenoid-like architecture of PL002-1792 [47]. This agreement is particularly important because structural prediction alone cannot confirm whether a recombinant protein adopts the predicted fold in solution [26]. The predominance of β-sheet structure and minimal α-helical content is also consistent with the secondary structural characteristics reported for the experimentally characterised ice-binding protein from *F. frigoris* (*FfIBP*), a representative bacterial β-solenoid ice-binding protein [42]. Therefore, the CD spectrum and secondary-structure deconvolution provide experimental validation of the AlphaFold3 model and strengthen the structural basis for the proposed antifreeze-like function of PL002-1792. Nevertheless, several limitations should be acknowledged. Circular dichroism spectroscopy reports the average secondary-structure composition of the protein population and therefore cannot confirm that every protein molecule attained the native conformation. Similarly, although SDS-PAGE indicated recovery of a predominantly monomeric protein, it cannot exclude the presence of minor populations of partially misfolded or structurally heterogeneous species. In addition, the refolding efficiency, protein recovery yield, and sample homogeneity were not quantitatively evaluated, as the primary objective of this study was to obtain sufficient soluble protein for structural and functional characterization. Future studies employing complementary biophysical techniques, such as size-exclusion chromatography, dynamic light scattering, or analytical ultracentrifugation, together with quantitative assessment of protein recovery, will provide a more comprehensive evaluation of refolding efficiency, aggregation state, and conformational homogeneity.

Functional analysis demonstrated that PL002-1792 possesses measurable ice recrystallisation inhibition activity (Figure 7). The ice recrystallisation inhibition (IRI) activity of PL002-1792 was concentration-dependent, with progressively greater inhibition observed at increasing protein concentrations. Compared with highly active antifreeze proteins from insects or fish, the IRI activity of PL002-1792 may be considered moderate [39,59]. However, bacterial antifreeze proteins frequently exhibit lower activity than their eukaryotic counterparts while still providing substantial physiological benefits [30,50]. In natural environments, moderate IRI activity may be sufficient when combined with complementary cold-adaptation mechanisms such as compatible solute accumulation, membrane remodeling, and extracellular polymeric substance production [12]. Therefore, the relatively modest IRI activity observed here should not be interpreted as evidence of limited biological relevance. One limitation of the present study is the absence of a well-characterised bacterial ice-binding protein as a positive control, which would have enabled direct comparison of the antifreeze activity of PL002-1792 under identical assay conditions. Nevertheless, bovine serum albumin (BSA) is widely used as a negative control in IRI assays to establish the baseline for ice crystal growth, and the consistent reduction in mean grain size relative to the BSA control demonstrates that PL002-1792 possesses measurable ice recrystallisation inhibition activity. Future studies comparing PL002-1792 directly with experimentally characterised bacterial ice-binding proteins, such as the ice-binding protein from *F. frigoris*, will provide a more comprehensive assessment of its relative antifreeze activity. Interestingly, despite its moderate IRI activity, PL002-1792 conferred a marked improvement in freeze–thaw survival, suggesting that inhibition of ice recrystallisation alone may not fully explain the observed cryoprotective effect. Bacterial freeze tolerance is a multifactorial process involving not only modulation of ice crystal growth but also protection against membrane damage, osmotic stress, and other freezing-induced cellular injuries. Although the present study did not investigate these additional mechanisms, expression of PL002-1792 may contribute to cellular protection through indirect effects beyond measurable IRI activity. Future studies examining membrane integrity, osmotic stress responses, and protein–membrane interactions will be necessary to elucidate the precise mechanisms underlying the enhanced freeze–thaw tolerance conferred by PL002-1792.

The freeze–thaw protection assay provided the strongest functional evidence supporting a cryoprotective role for PL002-1792. Recombinant *E. coli* expressing PL002-1792 consistently exhibited higher survival than empty-vector controls throughout repeated freeze–thaw cycles (Figure 8). Following the first freeze–thaw cycle, PL002-1792-expressing cells retained 81% viability compared with 50% for the control. After the second and third freeze–thaw cycles, survival remained relatively high at 70% and 67%, respectively, whereas viability of the control declined markedly to 37% and 27%. This represents an approximately 2.48-fold improvement in survival after the third freeze-thaw cycle and demonstrates that expression of PL002-1792 confers a measurable cellular advantage under freezing stress. The magnitude of protection exceeded that observed in the IRI assay alone, suggesting that the biological function of PL002-1792 may extend beyond simple inhibition of ice recrystallisation [57,59]. Potential mechanisms include stabilisation of extracellular ice interfaces, protection of membrane integrity, modulation of local ice crystal morphology, or interactions with other cellular stress-response systems [39,60]. Similar discrepancies between in vitro IRI activity and in vivo cryoprotective performance have been reported for other microbial antifreeze proteins, indicating that multiple mechanisms likely contribute to cellular protection [61,62].

The ecological significance of PL002-1792 likely extends beyond its moderate ice recrystallisation inhibition activity. Antarctic *Flavobacterium* species inhabit environments characterised by persistent subzero temperatures and recurrent freeze–thaw cycles, where limiting ice recrystallisation may reduce cellular damage and enhance survival [12,30]. Rather than functioning as an isolated adaptation, PL002-1792 is likely to act in concert with other established cold-adaptation mechanisms, including membrane remodelling, compatible solute accumulation, and extracellular polymeric substance production [12,50,60]. The strong transcriptional induction of PL002-1792 under severe subzero conditions further suggests that it contributes to an integrated physiological strategy for survival in polar environments.

**Figure 9:**
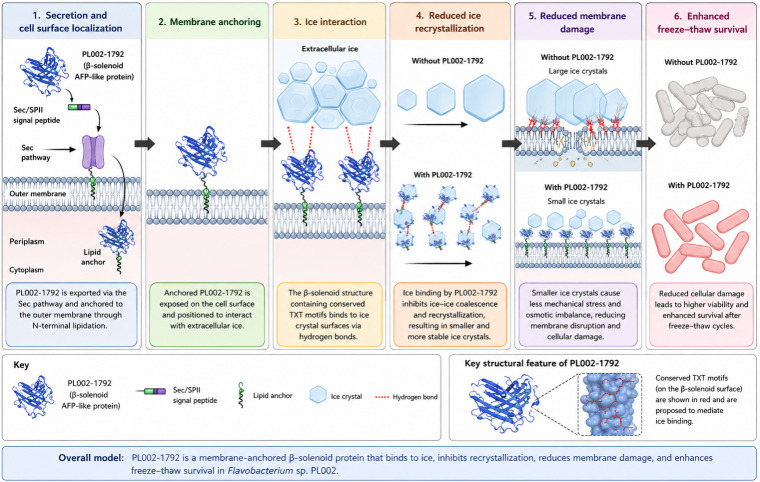
Proposed mechanism underlying the freeze-protective function of PL002-1792. The predicted Sec/SPII lipoprotein signal peptide directs PL002-1792 through the Sec secretion pathway, resulting in membrane anchoring at the bacterial cell surface. The β-solenoid structure containing conserved TXT-like motifs is proposed to interact with extracellular ice crystals, thereby inhibiting ice recrystallisation. Reduced ice crystal growth is hypothesised to minimise membrane damage and osmotic stress during freeze–thaw cycles, ultimately enhancing bacterial survival. The proposed mechanism is based on the combined computational and experimental evidence presented in this study and awaits further experimental validation. The schematic was created by the authors based on the findings of this study.

Based on the combined computational and experimental evidence, we propose that PL002-1792 contributes to freeze tolerance through a membrane-associated mechanism (Figure 9). The predicted Sec/SPII lipoprotein signal peptide suggests that the protein is exported and anchored to the cell surface, where its β-solenoid ice-binding architecture may facilitate interactions with extracellular ice crystals. By inhibiting ice recrystallisation, PL002-1792 may reduce membrane disruption and osmotic stress during repeated freeze–thaw cycles, thereby enhancing bacterial survival. Although this mechanistic model is supported by the structural predictions, ice recrystallisation inhibition assay, and freeze–thaw survival experiments, further studies are required to experimentally confirm protein localisation and the molecular basis of ice binding.

Collectively, the results support a model in which PL002-1792 functions as a cold-induced, surface-associated antifreeze-like protein that contributes to freezing tolerance in Antarctic *Flavobacterium* sp. PL002. The strong transcriptional induction under subzero conditions, the presence of a lipoprotein signal peptide, the β-sheet-rich ice-binding protein-like structure, measurable ice recrystallisation inhibition activity, and enhanced freeze–thaw survival all converge toward the same functional interpretation. More broadly, this work demonstrates the effectiveness of integrating transcriptomics, structural prediction, and experimental validation for the discovery of previously uncharacterized cold-adaptation proteins. As a substantial proportion of polar microbial genomes remains populated by hypothetical proteins, similar structure-guided approaches may reveal additional mechanisms that enable microbial survival in extreme cryogenic environments. Although this study is fundamentally focused on elucidating bacterial cold adaptation, the ice recrystallisation inhibition activity of PL002-1792 suggests potential for future development as a microbial-derived cryoprotective agent for frozen food preservation.

## 5. Conclusions

PL002-1792 was identified as a highly cold-induced hypothetical protein from Antarctic *Flavobacterium* sp. PL002 through transcriptomic analysis. Structural prediction revealed a β-sheet-rich architecture, a lipoprotein signal peptide, and conserved motifs commonly associated with bacterial ice-binding proteins. Experimental validation confirmed successful refolding of the recombinant protein into a predominantly β-sheet-rich structure. Functional assays demonstrated moderate ice recrystallisation inhibition activity and significantly enhanced freeze–thaw survival of recombinant *E. coli* expressing PL002-1792. These findings indicate that PL002-1792 functions as a novel antifreeze-like protein that contributes to cellular protection under freezing stress. Overall, this study highlights the effectiveness of integrating transcriptomics, structural prediction, and experimental validation to uncover the functions of previously uncharacterized proteins and advances our understanding of microbial cold adaptation in Antarctic environments.

## Figures and Tables

**Table 1:** Differential expression and annotation characteristics of the candidate cold-responsive gene PL002-1792 identified from *Flavobacterium* sp. PL002 under subzero growth conditions.

Gene ID	Comparison	Log_2_ Fold Change	Adjusted *p*-Value	Annotation	Best BLASTP Hit (% Identity)
PL002-1792	−20 °C vs. −6 °C	5.61	1.89 × 10^−138^	LIPL48 domain-containing protein	Hypothetical protein (*Flavobacterium muglaense*) (87.9%)
	−20 °C vs. 15 °C	4.89	1.10 × 10^−59^		

**Table 2:** Physicochemical properties of the candidate protein PL002-1792 predicted using the ExPASy ProtParam server.

Property	Value
Amino acid length	402 aa
Molecular weight	41.3 kDa
Theoretical pI	4.70
Instability index	12.66
Aliphatic index	75.02
GRAVY	−0.044

**Table 3:** Signal peptide prediction of PL002-1792 generated using SignalP 6.0.

Protein Type	Other	Signal Peptide (Sec/SPI)	Lipoprotein Signal Peptide (Sec/SPII)	TAT Signal (Tat/SPI)	TAT Lipoprotein Signal Peptide (Tat/SPII)	Pilin-like Signal Peptide (Sec/SPII)
Likelihood	0	0	1	0	0	0

**Table 4:** Structural validation of the AlphaFold3-predicted model of PL002-1792.

Validation Metric	Value
**AlphaFold3 pTM score**	0.96
**Ramachandran-favoured residues**	95.38
**Ramachandran-allowed residues**	4.62
**Ramachandran outliers**	0
**MolProbity score**	1.85
**Clashscore**	8.59
**Rotamer outliers**	0
**Verify3D**	91.98% (Pass, >80%)
**ERRAT**	90.33

The MolProbity score (1.85) and clashscore (8.59) correspond to the 83rd and 79th percentiles, respectively, relative to structures in the MolProbity reference database. A Verify3D score > 80% is generally considered indicative of a reliable three-dimensional model.

**Table 5:** Comparison of the predicted secondary structure composition of PL002-1792 with experimental CD spectroscopy.

Secondary Structure	AlphaFold3 (DSSP, %)	CD Spectroscopy (BeStSel, %)
**α-helix**	2.0	1.6
**β-sheet**	47.6	38.4
**Turn**	12.9	16.0
**Other/Coil**	37.5	44.0
**TOTAL**	100	100

## Data Availability

The original contributions presented in this study are included in the article. The RNA sequencing datasets generated in this study have been deposited in the NCBI Sequence Read Archive (SRA) under BioProject accession PRJNA1337565 and SRA accession SRR35731852. The genome sequence of *Flavobacterium* sp. strain PL002 is available in the NCBI GenBank database under accession GCF_054165825.1. The AlphaFold3 structural model (PDB format), circular dichroism (CD) spectroscopy data, and other supporting datasets generated during the current study are available from the corresponding author upon reasonable request.
